# Room‐Temperature Collective Quantum Emission Mediated by Wannier–Mott Excitons in CsPbBr_3_ Nanowires

**DOI:** 10.1002/smsc.202500400

**Published:** 2025-09-29

**Authors:** Mutibah Alanazi, Atanu Jana, Duc Anh Nguyen, Sangeun Cho, Sanghyuk Park, Hannu P. Pasanen, Oleksandr Matiash, Frédéric Laquai, Robert A. Taylor, Youngsin Park

**Affiliations:** ^1^ Physics Department, College of Science Jouf University, Al‐Jouf Sakaka P.O. Box 2014 Saudi Arabia; ^2^ Clarendon Laboratory Department of Physics University of Oxford Parks Road Oxford OX1 3PU UK; ^3^ Division of System Semiconductor College of AI Convergence Dongguk University Seoul 04620 Republic of Korea; ^4^ KAUST Solar Center Physical Sciences and Engineering Division Material Science and Engineering Program King Abdullah University of Science and Technology (KAUST) Thuwal 23955‐6900 Kingdom of Saudi Arabia; ^5^ Department of Chemistry LMU Münich Butenandtstraße 5‐13(E) D‐81377 München Germany; ^6^ Department of Chemistry College of Natural Science Ulsan National Institute of Science and Technology Ulsan 44919 Republic of Korea

**Keywords:** cesium lead bromide nanowires, collective quantum emissions, room‐temperature quantum optics, superfluorescence, Wannier–Mott excitons

## Abstract

Room‐temperature collective quantum emission (RT‐CQE), enabled by many‐body interactions and phase‐synchronized dipole oscillations, offers a promising path for scalable quantum photonics. Here, superfluorescence (SF) is demonstrated in CsPbBr_3_ perovskite nanowires (NWs), facilitated by Wannier–Mott excitons with spatially delocalized wavefunctions and strong dipole–dipole interactions. The intrinsic quasi‐1D geometry and occasional bundling promote preferential dipole alignment along the NW axis, enabling long‐range phase coherence. Key experimental signatures, photon bunching with *g* ^2^(0) ≈2, femtosecond‐scale coherence time (≈88 fs), and ultralow excitation threshold (≈210 nJ^−1^ cm^2^), confirm the onset of SF at ambient conditions. Ultrafast spectroscopy reveals bandgap renormalization, state filling, and exciton‐phonon coupling, consistent with collective excitonic behavior mediated by delocalized states. Unlike other RT‐SF mechanisms based on polarons or electron–hole liquids, the system exploits directional dipole alignment and exciton delocalization in quasi‐1D NWs, allowing coherent emission without the need for high excitation densities or complex structural ordering. These findings demonstrate that CsPbBr_3_ NWs can sustain RT‐SF driven by exciton delocalization and directional dipole coupling, providing a new physical platform for coherent light generation under ambient conditions.

## Graphical abstract

Room‐temperature collective quantum emission emerges in partially aligned CsPbBr_3_ nanowires through spontaneous synchronization of Wannier–Mott excitons. This alignment enables cooperative dipole–dipole interactions that result in coherent photon bursts (N^2^ × hν), even under ambient conditions. The emission shows clear superfluorescent signatures including threshold behavior, polarization anisotropy, and sub‐nanosecond temporal bunching.

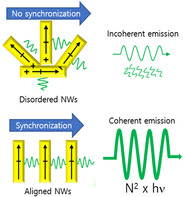

## 1 Introduction

Coherent light emission plays a central role in quantum technologies, enabling applications in quantum information processing, integrated photonics, and advanced optoelectronics.^[^ ^1^, ^2^ ^]^ Among various emission regimes, collective quantum optical phenomena such as superfluorescence (SF) and superradiance (SR) are distinguished by their ability to generate macroscopic coherence through many‐body interactions.^[^ ^3^, ^4^, ^5^ ^]^ These phenomena emerge when quantum emitters, such as excitons, interact via coherent dipole–dipole coupling, resulting in synchronized radiative decay that enhances both emission intensity and temporal coherence.^[^ ^6^, ^7^, ^8^ ^]^ However, such effects have traditionally been observed only at cryogenic temperatures, where thermal decoherence is suppressed.^[^ ^9^, ^10^, ^11^ ^]^ Realizing these collective emission processes under ambient conditions remains a critical challenge for practical and scalable quantum light sources.^[^ ^12^, ^13^, ^14^ ^]^ Metal halide perovskites (MHPs) have recently attracted attention as promising candidates for room‐temperature (RT) quantum photonics, owing to their tunable bandgaps, strong light‐matter coupling, and solution‐processable nature.^[^ ^15^, ^16^, ^17^, ^18^ ^]^ In particular, CsPbBr_3_ nanowires (NWs) offer a compelling platform, combining strong excitonic responses, anisotropic dipole alignment, and structural stability‐key ingredients for sustaining collective quantum emission (CQE) at RT.^[^ ^19^, ^20^, ^21^, ^22^, ^23^, ^24^ ^]^ Despite some reports of SF and SR in perovskite nanostructures at low temperatures, demonstrations of these effects under ambient conditions remain rare due to phonon‐mediated dephasing and thermal disruption of coherent dipole interactions.^[^ ^25^, ^26^, ^27^ ^]^ Theoretical models, including Dicke's SR formalism and the Maxwell–Bloch equations, describe how quantum emitters can synchronize via strong coupling, resulting in macroscopic coherence and cooperative decay dynamics.^[^ ^28^, ^29^, ^30^ ^]^ In contrast to organic semiconductors dominated by tightly bound Frenkel excitons, MHPs support Wannier–Mott excitons with spatially extended wavefunctions, moderate binding energies (≈20–50 meV), and large Bohr radii (≈4 nm).^[^ ^31^, ^32^, ^33^ ^]^ These features enable long‐range dipole–dipole interactions, making them suitable for sustaining coherent emission phenomena like SF. While CsPbBr_3_ quantum dots (QDs) retain Wannier–Mott excitonic character, their spatial confinement and large surface‐to‐volume ratio increase susceptibility to dephasing and nonradiative recombination, which hinder coherence and suppress collective emission, as evidenced by the necessity of ordered superlattices to observe SF.^[^ ^18^, ^34^ ^]^ In contrast, quasi‐1D CsPbBr_3_ NWs facilitate extended exciton delocalization and intrinsic dipole alignment along the wire axis, which promotes phase‐synchronized emission even at RT.^[^ ^19^, ^22^ ^]^ Recent studies have proposed various physical routes toward RT‐CQE in perovskite systems. For example, Biliroglu et al. observed RT‐SF in hybrid perovskites mediated by solitonic exciton‐lattice states stabilized by coherent polaron oscillations.^[^ ^35^ ^]^ Naresh et al. reported SF from an electron–hole liquid (EHL) phase in CsPbI_3_ films, requiring high excitation densities and exhibiting spectral features characteristic of plasma condensation.^[^ ^36^ ^]^ In contrast, our work presents a distinct excitonic mechanism based on spatially delocalized Wannier–Mott excitons in CsPbBr_3_ NWs, where long‐range dipole–dipole interactions and anisotropic alignment facilitate RT‐SF without invoking polaronic effects, soliton formation, or EHL condensation. Here, we demonstrate RT‐SF in CsPbBr_3_ NWs using a combination of micro‐photoluminescence (μPL), Hanbury Brown–Twiss (HBT) photon correlation, Michelson interferometry, and ultrafast transient reflectivity (TR) spectroscopy.

## 2 Results and Discussion

### 2.1 Structural Characterization and Dipole Alignment in CsPbBr_3_ NWs

**Figure 1:**
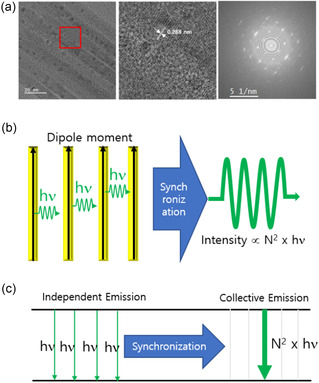
Structural characterization and dipole alignment in CsPbBr_3_ NWs. a) TEM images of CsPbBr_3_ NWs. The left panel shows a low‐magnification TEM image, highlighting well‐aligned NWs. The middle panel shows a high‐resolution transmission electron microscopy (HRTEM) image of the region indicated by the red box, revealing a lattice spacing of ≈0.288 nm corresponding to the (200) planes of cubic‐phase CsPbBr_3_. The right panel displays the corresponding SAED pattern, confirming the cubic perovskite crystal structure through characteristic diffraction rings/spots. Scale bars: 20 nm (left, middle), 5 1 nm^−1^ (right). b) Schematic illustration of dipole alignment and synchronization in NWs. Yellow rectangles represent individual NWs with black arrows indicating the direction of aligned dipole moments. Green sinusoidal waves show the emitted light, with synchronization (blue arrow) facilitating coherent CQE. c) Energy diagram comparing independent emission and CQE processes. The left side shows independent photon emission (*hν*), while the right side illustrates synchronized emission from N dipoles, producing a total emitted energy proportional to *N* × *h*ν and a peak intensity scaling as *N* ^2^. The notation “*N* × *hν*” indicates the total photon energy released in a single coherent emission event from *N* emitters and is a general feature of CQE processes, not exclusive to SR.

CsPbBr_3_ NWs were synthesized via a modified hot‐injection method, as described in the Methods section. **Figure** **1**a presents transmission electron microscopy (TEM) images of the resulting NWs. The low‐magnification image (left) reveals well‐aligned NWs with diameters of ≈ 10 nm, while the high‐resolution image (middle) shows a lattice spacing of ≈0.288 nm, corresponding to the (200) planes of cubic‐phase CsPbBr_3_. The corresponding selected area electron diffraction (SAED) pattern (right) shows distinct diffraction spots, confirming the single‐crystalline nature and high crystalline quality of individual NWs. This high degree of structural order is critical for supporting coherent dipole–dipole interactions underlying collective optical phenomena. Such an order enables quantum emitters (e.g., excitons) to oscillate synchronously with phase‐locked dipole moments‐a prerequisite for macroscopic coherence in SF and SR. Additional TEM analysis (Figure S1, Supporting Information) reveals morphological heterogeneity typical of solution‐processed growth, including both randomly oriented NWs and spontaneously bundled regions with 2–5 nm interwire separations. While this heterogeneity represents a limitation of our synthesis method, regions with partial NW alignment provide sufficient dipole coupling for collective emission, as confirmed by our optical measurements. Elemental analysis via energy‐dispersive X‐ray spectroscopy (EDX, Figure S2, Supporting Information) confirms the presence of Cs, Pb, and Br in the NWs. Complementary powder X‐ray diffraction (Figure S3, Supporting Information) validates the cubic CsPbBr_3_ crystal structure. Figure 1b schematically illustrates dipole alignment and collective emission, showing that the periodic lattice structure supports dipole orientation along the NW axis, consistent with prior reports of anisotropic excitonic behavior in CsPbBr_3_ NWs.^[^ ^23^, ^24^ ^]^ Theoretical studies further indicate that Wannier–Mott excitons in such structures possess spatially extended wavefunctions along the axis, enhancing long‐range dipole–dipole coupling.^[^ ^25^, ^35^, ^36^, ^37^ ^]^ The term long‐range dipole–dipole interaction refers to phase‐coherent coupling that originates from nm‐scale separations between adjacent NWs within a bundle, yet propagates coherently across the μm‐scale excitation volume. While individual NW‐to‐NW coupling occurs over nanometer distances (2–5 nm interwire spacing), the collective phase synchronization extends across the entire ≈1 μm confocal excitation spot, encompassing multiple NW bundles. This effective coherence range is two orders of magnitude larger than the exciton Bohr radius (≈7 nm) and NW diameter (≈10 nm), enabling macroscopic phase synchronization across the nanowire ensemble and supporting the observed CQE. Finally, Figure 1c conceptually depicts the transition from independent emission (*h*ν) to a synchronized CQE state (N × *h*ν), reflecting key characteristics of SR and SF.^[^ ^7^, ^26^ ^]^

### 2.2 Photoluminescence Characteristics of CsPbBr3 Nanowires

**Figure 2:**
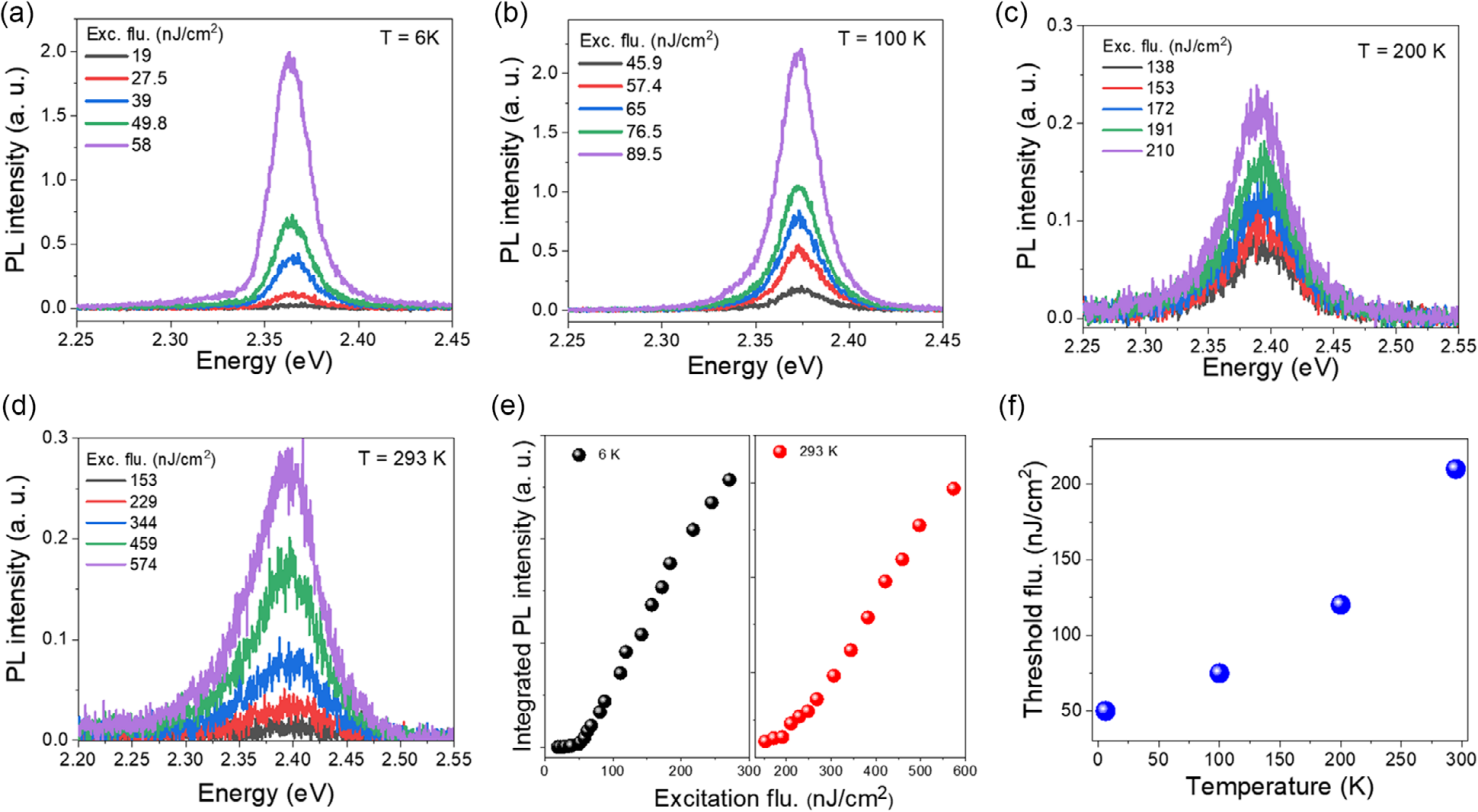
Excitation power‐dependent PL spectra of CsPbBr_3_ NWs measured at various temperatures. a) 6 K, b) 100 K, c) 200 K, and d) 293 K. Each spectrum is shown for different excitation fluences as indicated in the legends. e) Integrated PL intensity as a function of excitation fluence at 6 and 293 K, demonstrating a nonlinear dependence with a *P*_th_. f) Threshold fluence as a function of temperature, indicating that *P*_th_ increases with temperature. All the spectra were fitted using a Gaussian function (Figure S4, Supporting Information).

The emergence of RT‐CQE in CsPbBr_3_ NWs is strongly governed by excitation fluence (*P*) and temperature, which modulate the balance between dipole synchronization and dephasing mechanisms. To investigate these dependencies, we performed power‐dependent PL measurements at various temperatures (**Figure** **2**). At 6 K, a sharp nonlinear increase in PL intensity is observed at a threshold fluence of *P*_th_ = 50 nJ^−1^ cm^2^, signaling the onset of macroscopic dipole phase‐locking (Figure 2a). As the temperature increases, *P*_th_ systematically shifts to higher values: 75 nJ^−1^ cm^2^ at 100 K, 120 nJ^−1^ cm^2^ at 200 K, and 210 nJ^−1^ cm^2^ at 293 K (Figure 2b–d), consistent with enhanced thermal dephasing. Notably, the RT threshold (≈210 nJ^−1^ cm^2^) is significantly lower than previously reported values for SF, including Rainò et al. (3–5 μJ^−1^ cm^2^ at cryogenic temperatures)^[^ ^6^ ^]^ and Biliroglu et al. (≈5 μJ^−1^ cm^2^ at RT),^[^ ^7^ ^]^ highlighting the exceptionally efficient dipole coupling in CsPbBr_3_ NWs. This ultralow *P*_th_ likely arises from the NWs quasi‐1D geometry and high crystallinity, which promote directional dipole–dipole interactions and reduce inhomogeneous broadening, allowing cooperative emission to emerge even under weak excitation. The observed temperature dependence of *P*_th_ reflects a competition between quantum coherence and thermal decoherence: phonon suppression at low temperatures facilitates phase synchronization,^[^ ^5^, ^6^, ^7^ ^]^ whereas exciton‐phonon and carrier–carrier scattering at higher temperatures accelerate dephasing, requiring stronger excitation to sustain CQE.^[^ ^6^, ^27^, ^28^, ^32^ ^]^ To further quantify the transition into the collective regime, we analyzed the PL spectral linewidths using Gaussian fitting (Figure S4, Supporting Information). Across all temperatures, the full width at half maximum (FWHM) narrows near *P*_th_ (Figure S5, Supporting Information), consistent with a crossover from inhomogeneously broadened, localized emission to a delocalized, phase‐coherent regime.^[^ ^18^, ^36^, ^37^ ^]^ This spectral narrowing is a distinctive signature of synchronized dipole oscillations rather than conventional excitonic recombination.^[^ ^7^, ^30^, ^31^, ^32^, ^33^, ^34^, ^35^, ^36^, ^37^, ^38^ ^]^ In parallel, we fitted the integrated PL intensity (*I*_PL_) to a power‐law dependence, *I*_PL_α *P* ^*n*^, which reveals superlinear scaling (*n* > 2) above *P*_th_ (Figure S6, Supporting Information), a hallmark of cooperative quantum behavior.^[^ ^7^, ^30^, ^31^ ^]^ The power‐law exponent decreases from *n* = 4.3 at 6 K to *n* = 2.44 at 293 K, reflecting a gradual weakening of coherence with increased phonon interaction. However, even at 293 K, *n* remains significantly above 2, confirming that quantum coherence persists under ambient conditions. These observations align with the Wannier–Mott exciton model, wherein spatially delocalized excitons mediate long‐range dipole–dipole interactions along the NW axis.^[^ ^5^, ^14^ ^]^ In contrast to tightly bound Frenkel excitons in organic systems, the extended wavefunctions in perovskite NWs facilitate robust coherence and dipole alignment even at RT. All PL measurements were performed on the same CsPbBr_3_ NW ensemble using consistent excitation conditions. A fixed fluence of 382 nJ^−1^ cm^2^ was employed for temperature‐dependent spectra to maintain signal strength while avoiding thermal damage. The PL profiles remained spectrally consistent throughout thermal cycling (Figure S7, Supporting Information), confirming that the observed evolution arises from a uniform NW ensemble under well‐controlled conditions.

### 2.3 Polarization‐Resolved Photoluminescence and Dipole Alignment

**Figure 3:**
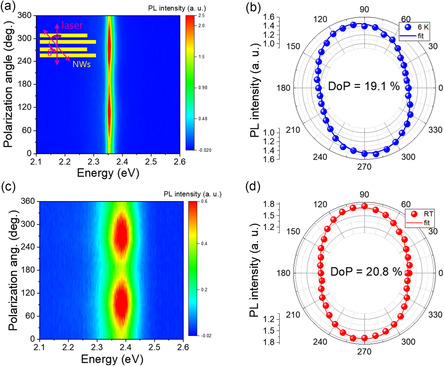
Polarization‐dependent PL of CsPbBr_3_ NWs. a) PL intensity map as a function of emission energy at 6 K. The inset schematically illustrates the definition of the polarization angle θ, which is the angle between the laser polarization direction (magenta arrow) and the NW axis (yellow bars). b) Polar plot of PL intensity versus polarization angle at 6 K. The blue dots represent experimental data, and the solid blue line is the fit. c) PL intensity map as a function of emission energy at RT. d) Polar plot of PL intensity versus polarization angle at RT. The red dots represent experimental data, and the solid red line is the fit.

To assess the directional coherence and dipole alignment in CsPbBr_3_ NWs, we performed polarization‐resolved PL measurements at 6 and 293 K (**Figure** **3**a–d). At 6 K, the PL spectrum exhibits a narrow, intense peak (Figure S8, Supporting Information), indicative of suppressed dephasing and high optical quality. In contrast, at 293 K, the emission broadens, consistent with enhanced thermal fluctuations and phonon‐induced dephasing. The measured degree of polarization (DoP) is essentially temperature‐independent within experimental uncertainty, 19.1% at 6 K and 20.8% at RT. This temperature‐independent DoP demonstrates that structural anisotropy and dipole alignment are preserved across the entire temperature range, while the observed degradation of collective emission signatures arises primarily from thermal dephasing effects rather than loss of orientational order. This stability suggests the persistence of structural and dipolar alignment even under ambient conditions, supporting the presence of coherent dipole–dipole interactions within the NW ensemble.^[^ ^5^, ^6^, ^29^ ^]^ The slight ellipticity observed in the polarization profiles may arise from quantum interference effects, where phase fluctuations between excitonic states modulate the emission polarization.^[^ ^28^, ^32^, ^33^ ^]^ Such features are consistent with previously reported nonlinear polarization dynamics in superfluorescent systems, where interemitter coupling and mesoscopic disorder can lead to deviations from ideal linear polarization.^[^ ^34^ ^]^ These experimental trends are also in line with Maxwell–Bloch models describing the temporal and polarization dynamics of synchronized dipole systems, which account for fluctuations in emission delay, peak intensity, and polarization state (see Supplementary Text).^[^ ^28^, ^33^, ^34^, ^35^, ^36^, ^37^, ^38^, ^39^, ^40^, ^41^ ^]^ Given the confocal excitation spot size (≈1 μm), the observed signal represents an ensemble average over multiple NW domains with various orientations. For purely random orientations, complete orientational averaging would yield DoP ≈0% due to statistical cancelation of individual anisotropies. However, the measured DoP (≈20%) is non‐negligible and represents a significant deviation from this zero expectation, implying incomplete dipole randomization and residual alignment across the ensemble. The consistent observation of ≈20% DoP across multiple measurement spots indicates correlated orientations beyond statistical fluctuations (expected DoP α 1/√N → 0% for large random ensembles), providing evidence for collective dipole synchronization. This quantitative observation suggests collective dipole interactions that partially overcome orientational disorder, consistent with both spontaneous alignment within NW bundles and preferential dipole orientation driven by crystal anisotropy and cooperative effects.^[^ ^42^ ^]^ While polarization alone does not constitute definitive evidence of SF, its persistence‐alongside threshold‐like behavior, photon bunching, power‐law scaling, and temperature‐dependent linewidth evolution‐provides complementary support for cooperative dipole alignment and macroscopic coherence consistent with CQE.

### 2.4 Coherence Dynamics of CsPbBr_3_ NWs Probed by Michelson Interferometry

**Figure 4:**
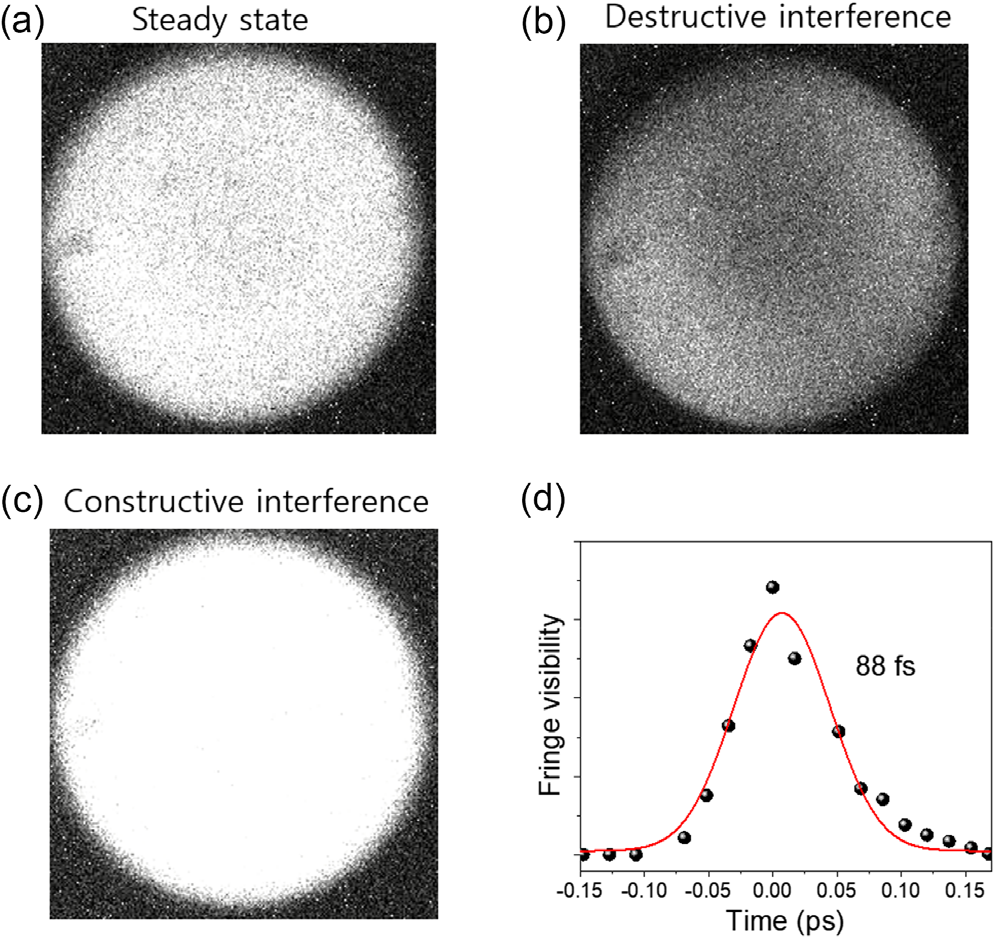
First‐order coherence of Michelson interferometer fringe patterns for CsPbBr_3_ NWs at RT. a) Steady state interference pattern showing the initial light intensity distribution. b) Destructive interference pattern, indicating areas of reduced intensity due to phase cancelation. c) Constructive interference pattern, demonstrating areas of increased intensity due to phase reinforcement. d) Fringe visibility as a function of time delay, showing a coherence time of ≈88 fs. The red curve represents a fit to the experimental data, illustrating the decay of fringe visibility with time delay.

To assess the temporal coherence of CsPbBr_3_ NWs under ambient conditions, first‐order quantum optical correlation measurements were performed using a Michelson interferometer.^[^ ^33^ ^]^ **Figure** **4**a–c shows representative interference patterns corresponding to steady‐state, destructive, and constructive interference, respectively. The gradual reduction in fringe visibility with increasing optical path difference reflects the finite coherence of the emitted light. The fringe visibility decay, plotted in Figure 4d and fitted with a Gaussian function, yields a coherence time (τ_c_) of ≈88 fs, consistent with previously reported values for perovskite nanomaterials.^[^ ^7^ ^]^ This relatively short coherence time is attributed to phonon‐mediated dephasing processes at RT.^[^ ^32^ ^]^ Nevertheless, the observation of well‐defined interference fringes confirms the presence of phase‐coherent emission, a key hallmark of CQE and SF.^[^ ^6^, ^43^ ^]^ The Gaussian profile of the visibility decay suggests ensemble emitter fluctuations, characteristic of SF dynamics, where emitter synchronization is subject to statistical variation.^[^ ^6^, ^43^ ^]^ The corresponding coherence length (L_c_ ≈ 26.4 μm), calculated from L_c_ = c·τ_c_, represents the free‐space propagation distance over which temporal coherence is maintained. In our quasi‐1D NW system, the effective coherent interaction volume is constrained by the ≈1 μm confocal spot size and NW bundle dimensions, rather than this theoretical propagation distance.^[^ ^33^ ^]^ These coherence properties can be directly linked to the intrinsic nature of Wannier–Mott excitons in CsPbBr_3_ NWs, in which their spatially delocalized wavefunctions support long‐range coupling, facilitating macroscopic phase coherence even under ambient conditions.

### 2.5 Second‐Order Correlation and Time‐Resolved Photoluminescence Measurements

**Figure 5:**
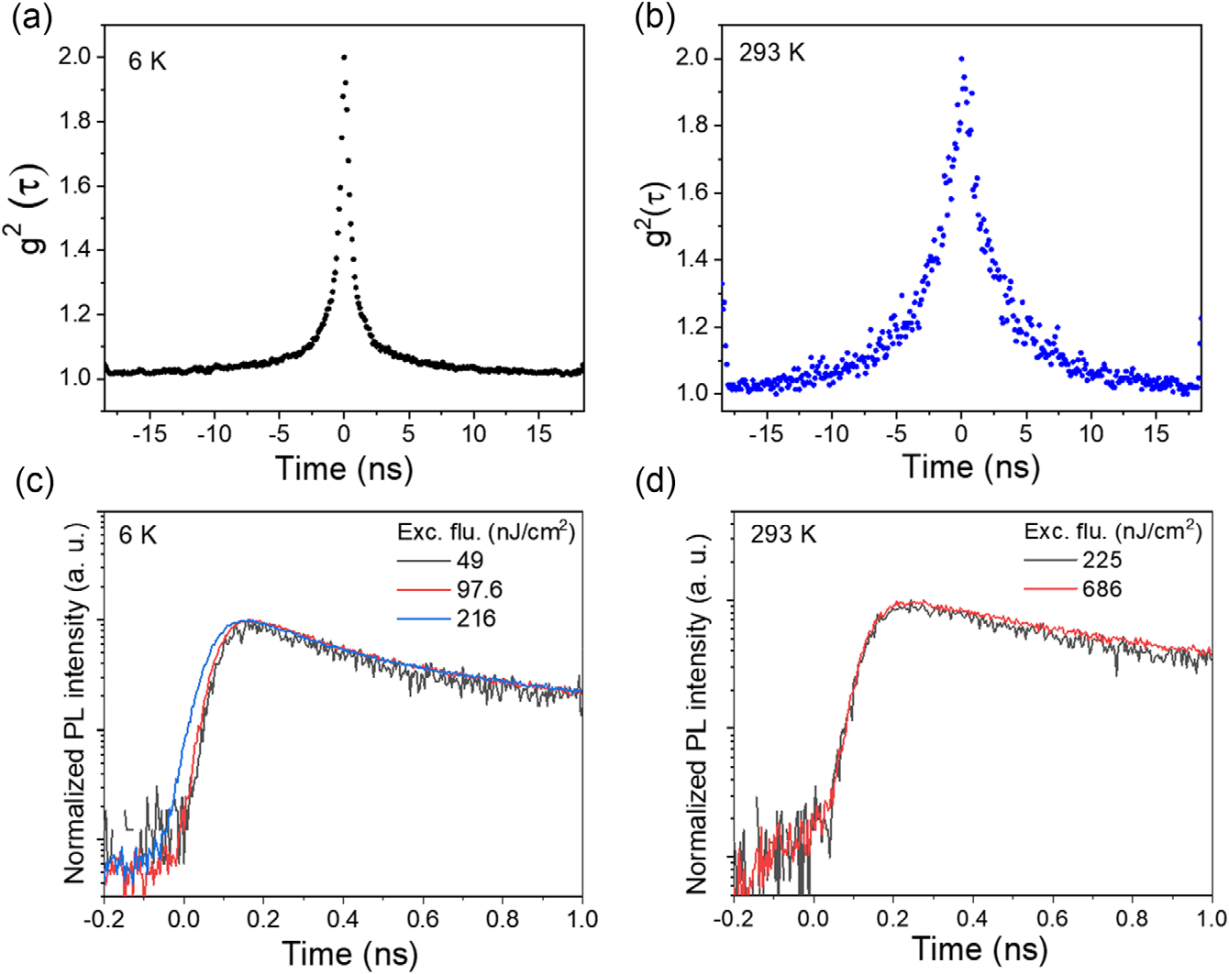
Second‐order correlation function g^2^(τ) and TRPL of CsPbBr_3_ NWs at 6 and 293 K. a) *g* ^2^(τ) at 6 K shows a narrow peak with a temporal width of ≈1 ns, indicating robust photon bunching. b) At 293 K, the *g* ^2^(τ) peak broadens to ≈3 ns, attributed to increased phonon interactions and thermal fluctuations. c) Time‐resolved PL at 6 K under varying excitation fluences, showing a fluence‐dependent delay in the initial rise. The delay is instrument‐limited and not conclusively attributed to SF buildup. d) At 293 K, TRPL measurements show a reduced coherence time and broader emission dynamics. The data collectively indicate that while SF is prominent at 6 K, collective emission phenomena persist at 293 K, albeit modified by thermal effects.

To further validate the presence of collective emission in CsPbBr_3_ NWs, we performed second‐order photon correlation *g* ^2^(τ) and time‐resolved PL (TRPL) measurements at 6 and 293 K (**Figure** **5**). The quantum optical properties of light can be characterized by correlation functions, where the first‐order correlation function *g* ^1^(τ) describes the field coherence (measured through interferometry as discussed previous), while the second‐order correlation function *g* ^2^(τ) reveals photon statistics and distinguishes between classical and quantum emission regimes (see Supplementary Text). For classical chaotic light, these correlations are connected via the Siegert relation: *g* ^2^(τ) = 1 + |*g* ^1^(τ)|^2^. Deviations from this relation, particularly values of *g* ^2^(0) approaching 2, are indicative of collective quantum effects such as SF. The CsPbBr_3_ NWs exhibit pronounced photon bunching with *g* ^2^(0) ≈2 at both temperatures (Figure 5a,b and Figure S9, Supporting Information), confirming the collective nature of the emission, such as SF, consistent with previous studies.^[^ ^7^ ^]^ The temporal width of the *g* ^2^(τ) peak increases from ≈1 ns at 6 K to ≈3 ns at 293 K, attributed to enhanced phonon interactions and thermal fluctuations. At 6 K, we observe an initial delay of ≈100 ps in the PL rise (Figure 5c), which gradually shortens to ≈50 ps with increasing excitation fluence. However, our time‐correlated single photon counting (TCSPC) system has a temporal resolution limit of ≈50 ps, which precludes definitive observation of classical SF build‐up dynamics. The absence of well‐resolved buildup delay and Burnham–Chiao ringing represents a limitation in our SF assignment, though the combination of other collective emission signatures suggests cooperative behavior. The observed delay may instead reflect a combination of instrument response, carrier thermalization, or propagation effects, and thus we present it as a tentative feature potentially consistent with collective behavior, but not conclusive. In contrast, no buildup delay is observed at 293 K (Figure 5d), suggesting that thermal dephasing limits the formation of a macroscopic coherent dipole. This behavior points to a transition from SF to a damped SF or amplified spontaneous emission (ASE) regime, where the coherence time (*T*_2_) becomes shorter than the critical threshold T_2_ < $\sqrt{\tau_{D}\tau_{r}}$, thereby inhibiting CQE. Additional factors such as propagation delay (τ_E_ = *L*/c) and quantum noise may also obscure delay signatures at RT.^[^ ^28^, ^44^, ^45^, ^46^, ^47^ ^]^ This transition can be attributed to the intrinsic properties of Wannier–Mott excitons in CsPbBr_3_ NWs, whose spatially extended wavefunctions and high oscillator strength facilitate the long‐range dipole–dipole interactions essential for SF at low temperatures. However, their delocalized nature also renders them susceptible to phonon‐induced dephasing at RT, leading to a suppression of coherent emission under ambient conditions. Biexponential fits to TRPL decay curves further support this interpretation. At 6 K, a dominant fast component (τ_1_ = 266 ps, A_1_ = 0.71) reflects collective emission, while a slower component (τ_2_ = 1.70 ns, A_2_ = 0.22) corresponds to spontaneous processes. At 293 K, the fast decay becomes slower (τ_1_ = 469 ps, A_1_ = 0.66), and the slow component becomes more pronounced (τ_2_ = 3.12 ns, A_2_ = 0.27), indicating a shift toward damped SF or ASE. Thermal lattice fluctuations at RT reduce dipole alignment and coherence time, limiting macroscopic polarization buildup and collective decay. In contrast, at low temperatures, quantum delocalization and enhanced radiative recombination driven by the giant oscillator strength effect reinforce coherent emission.^[^ ^48^, ^49^ ^]^ Together, these results demonstrate a temperature‐dependent crossover from SF to damped SF/ASE, governed by the coherence properties of Wannier–Mott excitons in CsPbBr_3_ NWs. CQE signatures were observed only in regions where NWs exhibited partial alignment or μm‐scale bundles, as shown in Figure 1. In contrast, isolated NWs with separations much greater than the exciton Bohr radius have not shown measurable first‐order coherence in previous reports. This indicates that nm‐scale separations within bundles, enabling effective coupling over μm distances, are essential for sustaining RT‐SF in this system.

### 2.6 Ultrafast Carrier Dynamics in CsPbBr_3_ NWs at RT

**Figure 6:**
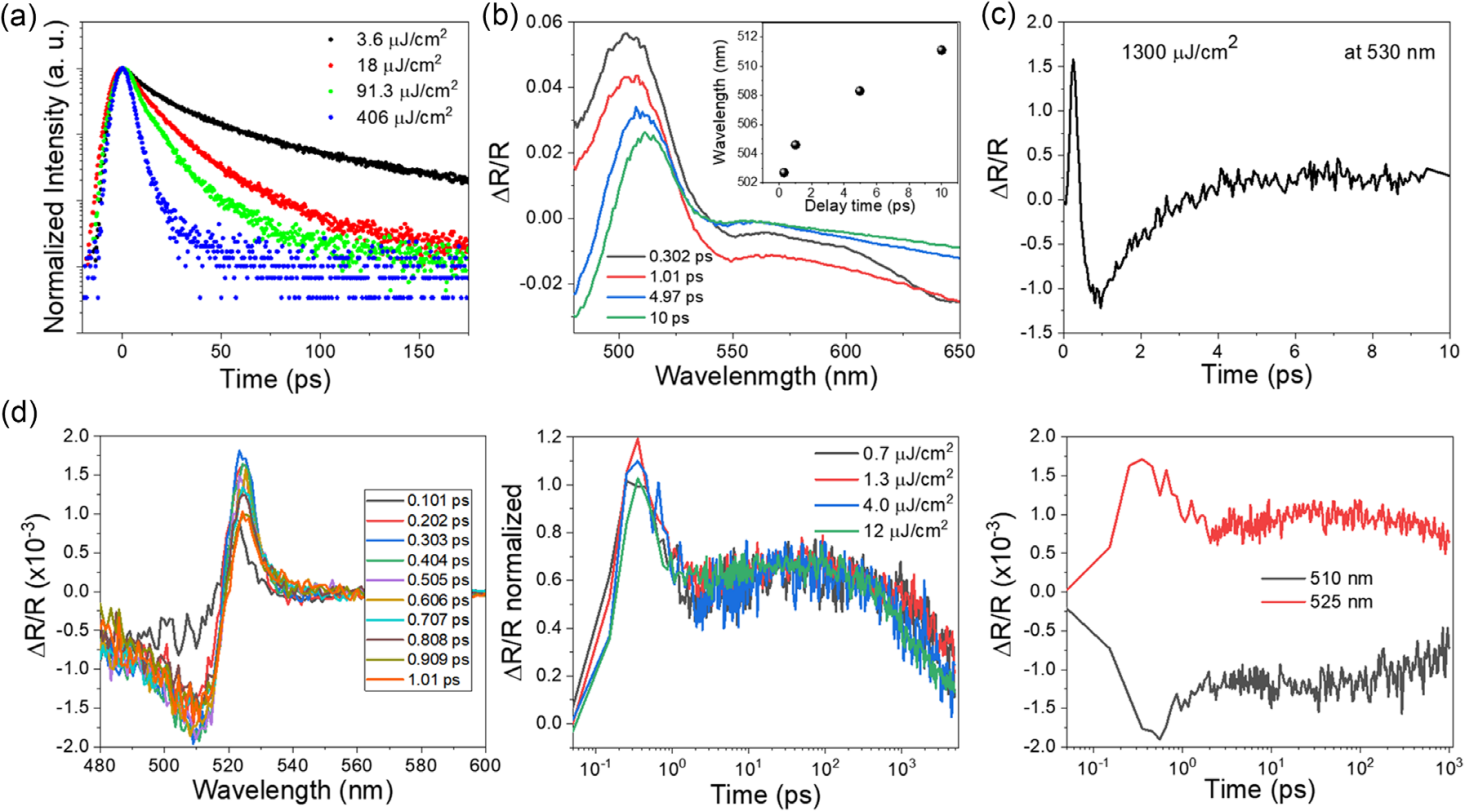
Ultrafast carrier dynamics of CsPbBr_3_ NWs at RT. a) Fluence‐dependent TRPL decay profiles measured at low (3.6 μJ^−1^ cm^2^) and high fluences (406 μJ^−1^ cm^2^), revealing accelerated recombination indicative of CQE at higher excitation densities. b) Wavelength‐dependent TR spectra at high excitation fluence of 1300 μJ^−1^ cm^2^, showing initial state filling (positive peak near 500 nm), followed by BGR‐induced redshift and negative Δ*R* from carrier‐induced absorption beyond 550 nm. c) Temporal dynamics of TR at moderate fluence, illustrating early‐stage Pauli blocking (positive peak at ≈520 nm) and subsequent bleaching and carrier‐induced absorption effects. d) Detailed TR spectra at moderate fluence, presented as three panels: (left) initial TR spectra indicating photoinduced state filling (≈520 nm); (middle) intermediate spectra with negative features (>530 nm) due to carrier‐induced absorption and bleaching; (right) long‐time spectra showing persistent signal near 525 nm, indicating stimulated emission or CQE.

**Figure** **6** presents the ultrafast carrier dynamics of CsPbBr_3_ NWs at RT, investigated through TRPL using a streak camera and TR spectroscopy. TRPL measurements (Figure 6a) show fluence‐dependent acceleration of decay dynamics, indicating a transition from isolated exciton recombination to CQE. At low fluence (3.6 μJ^−1^ cm^2^), the decay time is ≈150 ps, which shortens to ≈50 ps at high fluence (406 μJ^−1^ cm^2^), with a threshold near 90 μJ^−1^ cm^2^, consistent with the onset of SF as predicted by Maxwell–Bloch formalism.^[^ ^44^ ^]^ Although classical Burnham–Chiao ringing is not observed‐likely due to limited coherence length and phonon‐induced dephasing the observed dynamics support CQE behavior.^[^ ^50^ ^]^ Spectrally resolved TR data (Figure 6b–d) further elucidate these effects. At early time delays (≈0.3 ps, Figure 6b), a positive Δ*R* near 500 nm arises from state filling, which redshifts to ≈510 nm due to BGR, while negative Δ*R* beyond 550 nm indicates carrier‐induced absorption and excited‐state transitions.^[^ ^51^, ^52^ ^]^ At high fluence (Figure 6c), pronounced many‐body interactions, including exciton‐exciton coupling and refractive index modulation, manifest as rapid reflectivity dips (≈1 ps). Recovery dynamics between 1–10 ps reflect carrier–carrier scattering and phonon relaxation processes. At moderate fluence levels (Figure 6d), spectra show positive Δ*R* near 520 nm due to Pauli blocking, followed by negative features beyond 530 nm attributed to excited‐state absorption and bleaching.^[^ ^50^, ^52^ ^]^ These observations are closely aligned with the physics of Wannier–Mott excitons in CsPbBr_3_ NWs. The spatially extended wavefunctions and high oscillator strengths of these excitons facilitate long‐range dipole–dipole interactions and strong many‐body coupling‐hallmarks of CQE.^[^ ^53^, ^54^ ^]^ State filling, BGR, and Pauli blocking are characteristic excitonic effects in high‐density Wannier–Mott regimes.^[^ ^51^, ^52^, ^53^, ^54^, ^55^ ^]^ Moreover, the fluence‐dependent threshold behavior and accelerated recombination dynamics further support phase synchronization enabled by excitonic delocalization. The absence of ringing does not contradict SF but reflects short coherence lengths and fast dephasing, consistent with coherent emission sustained by Wannier–Mott excitons.^[^ ^35^, ^36^, ^37^, ^50^, ^53^ ^]^ Collectively, these findings provide compelling evidence for RT‐CQE in CsPbBr_3_ NWs and highlight their potential as scalable quantum light sources based on exciton‐mediated collective emission.^[^ ^7^, ^55^ ^]^

## 3 Conclusion

We demonstrate that CsPbBr_3_ NWs support RT‐CQE via SF driven by delocalized Wannier–Mott excitons. Their anisotropic structure enables dipole alignment and long‐range interactions, yielding macroscopic phase coherence under ambient conditions. Key signatures, including photon bunching *g* ^2^(0) ≈2, femtosecond coherence, and low excitation thresholds, confirm coherent light emission. Ultrafast spectroscopy further supports the delocalized exciton model through observations like state filling and BGR. These results establish CsPbBr_3_ NWs as strong candidates for CMOS‐compatible quantum photonic circuits, with future work needed in nanowire placement, optical coupling, and stability for on‐chip, RT light sources.

**Scheme 1:**
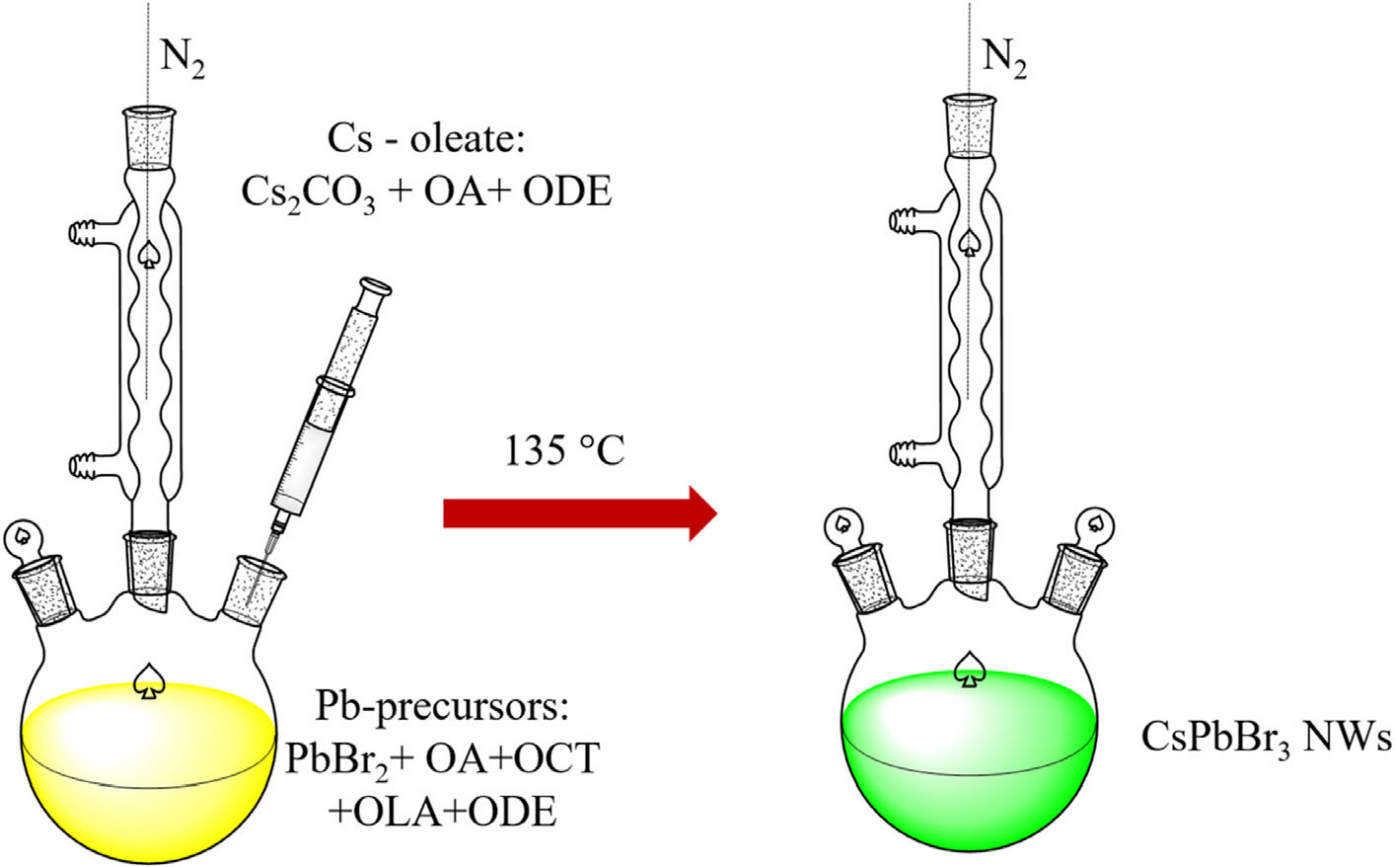
Scheme for the synthesis of CsPbBr_3_ NWs. The process begins with the preparation of Cs‐oleate by reacting Cs_2_CO_3_, oleic acid, and octadecene under an inert nitrogen atmosphere. Once the Cs‐oleate is prepared, it is injected into a lead precursor solution at 135 °C, containing lead bromide, octylamine, oleylamine, oleic acid, and other solvents such as octadecene and lauric acid. This reaction results in the formation of CsPbBr_3_ NWs as the final product.

## 4 Experimental Section

4.1

4.1.1

##### *Synthesis Methods of CsPbBr*_*3*_ *Nanowires*

In a 50 mL round‐bottom flask, 0.2 g of Cs_2_CO_3_ (99%) was combined with 625 μL of oleic acid (OA; 90%) and 7.5 mL of octadecene (ODE; 90%). The mixture was degassed under vacuum and dried at 120 °C for 1 h. In a 3‐neck flask, 5 mL of ODE and 0.2 mmol of lead bromide (PbBr_2_) (≥98%) were degassed under vacuum at 120 °C for 20 min. Under a nitrogen atmosphere, 0.8 mL of octylamine (OCT; 99%) was added, causing the solution to turn milky white. Then, 0.8 mL of oleylamine (OLA; 70%) was added, briefly making the solution transparent before it returned to a milky white state. The mixture was heated to 135 °C for 20 min. Afterward, 0.7 mL of the prepared Cs‐oleate solution was quickly injected. The reaction was quenched immediately by cooling with cold water. The CsPbBr_3_ NW s were isolated by centrifugation at 6000 rpm for 5 min and washed with hexane. Finally, CsPbBr_3_ NW s in a toluene solution were dispersed on Au‐patterned 300 nm‐SiO_2_ substrates. The Au‐patterned substrates were primarily used as positional markers to aid in locating dispersed CsPbBr_3_ NWs, rather than to actively induce alignment. The detailed synthesis process was depicted in **Scheme** **1**.

The NWs were deposited via drop‐casting, and no deliberate alignment method (e.g., flow‐assisted assembly or surface patterning) was employed. Nevertheless, spontaneous alignment of small NW bundles was occasionally observed, likely due to solvent evaporation effects or weak van der Waals interactions during deposition. Due to the extremely small NW diameter (≈10 nm) and the diffraction‐limited excitation spot (≈1 μm), it was not possible to optically resolve individual NW orientations during μPL or TRPL measurements. As a result, the measurement area typically encompassed ensembles of NWs with both random and partially aligned orientations.

##### Structural Characterization

TEM images were taken on a JEOL JEM‐2100F electron microscope using a 200 kV electron to analyze the morphology and size distribution of the CsPbBr_3_ NWs. Samples were prepared on 200‐mesh carbon‐coated Cu grids by dropping dilute nanowire solutions, which were then allowed to evaporate. Powder X‐ray diffraction measurements were performed on dried CsPbBr_3_ NW samples using Cu Kα radiation. The NW samples were prepared as powder by collecting the precipitated NWs from the solution synthesis. Diffraction patterns were collected over the 2*θ* range of 10°–50° with a step size of 0.02°. Due to the limited sample quantity available from NW synthesis, the diffraction intensities are relatively low but sufficient for phase identification.

##### Optical Measurement Experimental Setup: Photoluminescence

A pulsed NKT fiber laser with a tunable filter was used as the excitation source, providing output between 450 and 500 nm. The incident laser power on the CsPbBr_3_ surface ranged from a few nW to a few hundred of nW. The sample was mounted in a continuous‐flow helium cryostat, allowing the temperature to be controlled accurately from 6 K to RT. The optical properties were characterized using a confocal μPL setup. A 100× objective was held by a submicron precision piezoelectric stage above the cryostat and used to focus the incident laser beam to a spot size of ≈1 μm^2^ and to collect the resulting luminescence. The laser beam is focused onto the sample using a microscope objective lens, which ensures that a small, well‐defined area of the sample is illuminated. The luminescence was then directed to a 0.5 m focal length spectrometer with a 300 g mm^−1^ grating. The signal was finally detected using a cooled charge‐coupled device (CCD) detector. A telecentric lens arrangement was also present, allowing the incident angle of the exciting laser at the entrance to the objective to be varied by a computer‐controlled mirror thus providing an independent means to move the exiting spot relative to the collected emission, which is imaged confocally through the center of the objective. To ensure systematic comparison across the full temperature range (6–293 K), identical measurement parameters including laser power, integration time, spectrometer resolution, and detection sensitivity, were maintained for all temperature‐dependent measurements. The excitation laser was linearly polarized, and polarization‐resolved PL measurements were performed using a rotating linear analyzer positioned in the detection path before the spectrometer. The PL intensity was recorded as a function of analyzer angle (0° to 360°) to determine the DoP. Due to the synthesis and deposition method, the CsPbBr_3_ NWs exhibit a naturally random orientational distribution across the substrate surface, as confirmed by TEM imaging. Each measurement spot (≈1 μm diameter) encompasses multiple NW bundles oriented at various angles relative to both the excitation polarization and detection axis. This random distribution results in ensemble averaging over all possible orientations between the laser polarization and individual nanowire axes.

##### Optical Measurement Experimental Setup: HBT Experiment

The HBT measurements were carried out using the same experimental setup as above. The dispersed PL was reflected toward a photomultiplier connected to a commercial photon counting system. Measurements of the lifetimes of the confined states were then carried out over a range of excitation power densities. The HBT experiment setup using TCSPC and avalanche photodiodes (APDs), having a temporal resolution of 50 ps, is designed to measure the second‐order correlation function g^2^(τ) with a 50% fiber splitter coupling the emission from the spectrometer into the APDs. This measurement provides insights into the statistical properties of the emitted photons, such as photon bunching and coherence. The TCSPC module records the time differences between detection events in the two APDs. This data is used to construct a histogram of coincidence counts.

##### Optical Measurement Experimental Setup: Michelson Interferometer Experiment

A Michelson interferometer is a versatile optical setup commonly used to measure the coherence properties of light, including PL from nanomaterials like CsPbBr_3_ NWs. A 50:50 beam splitter is used to divide the PL into two paths. This beam splitter also recombines the two beams after reflection from the mirrors. One of the split beams is directed toward a fixed reference mirror. The other beam is directed toward a movable mirror. The position of this mirror can be adjusted precisely using a motorized translation stage, which allows for varying the path length of this beam. The two beams, after reflecting off their respective mirrors, are recombined at the beam splitter. The recombined light then travels to the detector, creating an interference pattern. The path length difference between the two beams can be adjusted by moving the movable mirror, which affects the interference pattern and allows for the measurement of coherence. The recombined light (interference pattern) is directed toward a photodetector, such as a CCD camera. The coherence length and time of the emitted PL are determined by analyzing the visibility of the interference fringes. High visibility indicates high coherence, whereas reduced visibility indicates dephasing and loss of coherence.

##### Optical Measurement Experimental Setup: Ultrafast Spectroscopy Measurements

TR spectroscopy was carried out using a home‐built pump‐probe setup at RT. The output of a Ti:Sapphire amplifier (Coherent LEGEND DUO, 800 nm, 4.5 mJ, 3 kHz, 100 fs) was split into a pump and a probe beam. The pump beam was directed to an optical parametric amplifier (OPA; Light Conversion TOPAS Prime with NIRUVIS extension) to produce 450 nm pulses, and mechanically chopped to 1500 Hz. The probe beam was focused onto a continuously moving CaF_2_ crystal for white light generation in visible range, or onto a sapphire window for the near‐infrared. The pump‐probe delay time was achieved by varying the probe path length using a broadband retroreflector mounted on a 600 mm automated mechanical delay stage (Thorlabs optical delay line ODL600/M), generating delays from −400 ps to 7 ns. The samples were measured in a nitrogen atmosphere at RT. The pump spot size with a 0.8 mm diameter was estimated from a Gaussian fit at 86.5% intensity. The pump spot Gaussian peak where the probe is focused onto has approximately two times more intensity than the average intensity, and therefore, the measured pump power is multiplied by two.

TRPL spectra were also collected using a free‐space coupled streak camera setup. A wavelength‐tunable Ti:Sapphire oscillator laser‐CHAMELEON ULTRA I from Coherent, emission wavelength of 900 nm, repetition rate of 80 MHz ‐ was coupled to an optical parametric oscillator (Coherent Compact OPO), generating a second harmonic beam of 450 nm. The sample was extracted from the film side. The laser spot size was set to roughly 45 × 55 μm. Samples were kept under a dynamic vacuum of <10^−4^ mbar. A custom‐built system of lenses was used to collect the emission at the entrance slit of a spectrograph (Princeton Instruments Acton Spectra Pro SP2300), and detected with a streak camera (Hamamatsu C10910) system in its Synchroscan unit configuration, using proprietary software (HPDTA). Laser excitation was filtered out using a 480 nm long‐pass filter.

## Supporting Information

Supporting Information is available from the Wiley Online Library or from the author.

## Conflict of Interest

The authors declare no conflict of interest.

## Supporting information

Supplementary Material

## Data Availability

The data that support the findings of this study are available from the corresponding author upon reasonable request.
