# Supplementary material for: Room‐Temperature Collective Quantum Emission Mediated by Wannier–Mott Excitons in CsPbBr3 Nanowires
Source: Small Sci. 2025 Sep 29;5(11):2500400. doi: 10.1002/smsc.202500400 (PMC12622410; doi:10.1002/smsc.202500400)
Supplement: Supplementary file 1 — Supplementary Material [file SMSC-5-2500400-s001.pdf]

Supporting Information

Room-temperature Collective Quantum Emission Mediated by Wannier–Mott Excitons in $CsPbBr_3$ Nanowires

Mutibah Alanazi, Atanu Jana, Duc Anh Nguyen, Sangeun Cho, Sanghyuk Park, Hannu P. Pasanen, Oleksandr Matiash, Frédéric Laquai, Robert A. Taylor, and Youngsin Park

Supplementary Text

Maxwell-Bloch equations

The Maxwell-Bloch equations may explain the intrinsic features of spontaneously emitted superfluorescent pulses in a system involving of multiple initially excited two-level atoms. The SF pulse is initiated by random initial polarizations caused by quantum fluctuations. The radiated fields resulting from various initial polarization distributions exhibit significant variations from pulse to pulse. These variations illustrate the amplification of quantum uncertainties to the macroscopic level[1]

$$\frac{\mathrm{d}P}{\mathrm{d}t} = \frac{-i}{\mathrm{h}}[P(t), H] - \gamma P(t) \qquad (1)$$

in which, $(\frac{\mathrm{d}P}{\mathrm{d}t})$ is the polarization rate over time, $(\frac{-i}{\mathrm{h}})$ is a quantum dynamic factor, $[P(t), H]$ is the commutators of polarization with H, $(\gamma P(\mathrm{t}))$ is the damping phase of polarization due to the interaction with surround medium. Therefore, the quantum dynamics of this system describe by four elements: photon density, photon-assisted polarization, normalized population in the excited state and the correlation between polarizations in two level systems (TLSs).

$$H = \sum_{l=1}^{N=\mathrm{tot}} \hbar\omega_l a_l^\dagger a_l + \int \mathrm{d}k \hbar\omega_k^{\mathrm{P}} b_k^\dagger b_k - i\hbar \sum_{l=1}^{N=\mathrm{tot}} \mathrm{d}k G(\omega_k^{\mathrm{P}})(a_l^\dagger b_k - b_k^\dagger a_l) \quad (2)$$

where the first, second, and third terms are the energies of the TLSs ensemble, the photon system, and the interaction between TLSs and photons, respectively. The terms $\hbar\omega_l$ and $\hbar\omega_k^{\mathrm{P}}$ are the energies of the l-th TLSs and a photon, respectively, where ω_k^{P} = c |k|, c is the speed of light, and k is a wave number of photons. The terms $a_1^\dagger(a_1)$ and $b_1^\dagger(b_1)$ are the creation

(annihilation) operators of TLSs and photons, respectively. The interaction coefficient $G\left(\omega_k^{\mathrm{P}}\right)$ is defined from the radiative lifetime of the single TLS τ_1,

$$G\left(\omega_k^{\mathrm{P}}\right) = \left[\frac{c^3}{8\pi\omega_0\omega_k^{\mathrm{P}}\, t_1}\right]^{1/2} \qquad (3).$$

Classical and quantum state of light

Coherence provides information on light, source size, emission processes, and light-matter interaction, including coherence time, spatial coherence, thermal or chaotic emission, and scattered light by atoms or molecules. These coherence properties can be described in a variety of methods. The temporal field correlation function $g^1(\tau)$, also known as the first-order correlation function, is one of the most frequently employed quantities.[2,3] It is defined as

$$g^1(\tau) = \frac{\langle E^*(t)E(t+\tau)\rangle}{\langle I(t)\rangle}$$

The intensity associated with the field E(t) is depicted by $I(t) = E^*(t)E(t)$, while the averaging over time t is represented by $\langle \ldots \rangle$. For example, a Michelson interferometer can be used to determine the temporal correlation function and other quantities associated with the first-order correlation function. The coherence properties of a light source can be fully characterized by measuring correlation functions at all orders, including the second-order temporal correlation function.[3]

$$g^2(\tau) = \frac{\langle I(t)\, I(t+\tau)\rangle}{\langle I(t)\rangle^2}$$

For chaotic light, or more broadly for fields exhibiting the properties of Gaussian processes, the correlation functions $g^n(\tau)$ are interconnected in a straightforward manner. The second-order correlation function $g^2(\tau)$ is linked to the modulus of the temporal first-order correlation function $g^1(\tau)$ in spatially coherent polarized chaotic light by Siegert relation[2]:

$$g^2(\tau) = 1 + |g^1(\tau)|^2$$

At zero delay, $g^2(0) > g^2(\tau \rightarrow \infty) = 1$, indicating an excess of intensity correlation, which is photon bunching.[2,4,5] In quantum optics, it is generally known that the Siegert relation's validity is not always guaranteed. For instance, the intensity fluctuations in laser light are primarily due to shot noise, resulting in a flat uncorrelated intensity correlation function. For this reason, measuring $g^2(\tau)$ enables the classification and discrimination of various sources and opens the door to the study of quantum effects.

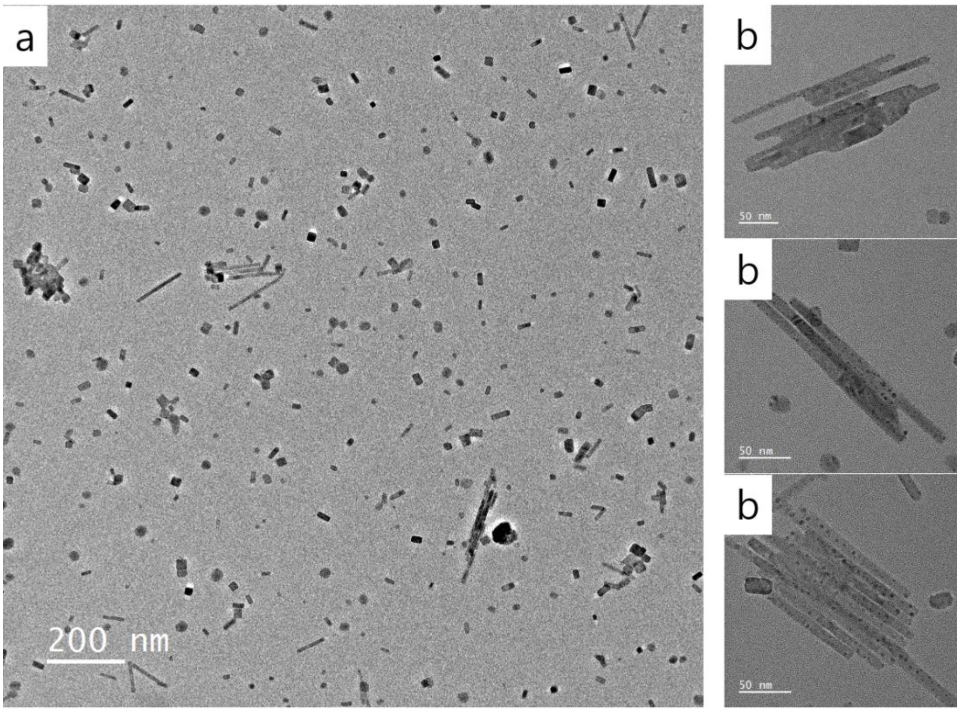

Figure S1. Transmission electron microscopy (TEM) images of the $CsPbBr_3$ NW ensemble prepared under the same synthesis and deposition conditions as used for optical measurements. (a) Low-magnification TEM image showing a heterogeneous distribution of nanowires, including both randomly oriented NWs and spontaneously formed aligned bundles. Scale bar: 200 nm. (b-d) High-resolution TEM images highlighting bundled regions with inter-nanowire separations on the order of 2-5 nm. Scale bars: 50 nm. These structural features indicate the presence of partially ordered domains within the ensemble, providing a realistic platform for exciton coupling via dipole–dipole interactions and supporting the collective emission behavior discussed in the main text.

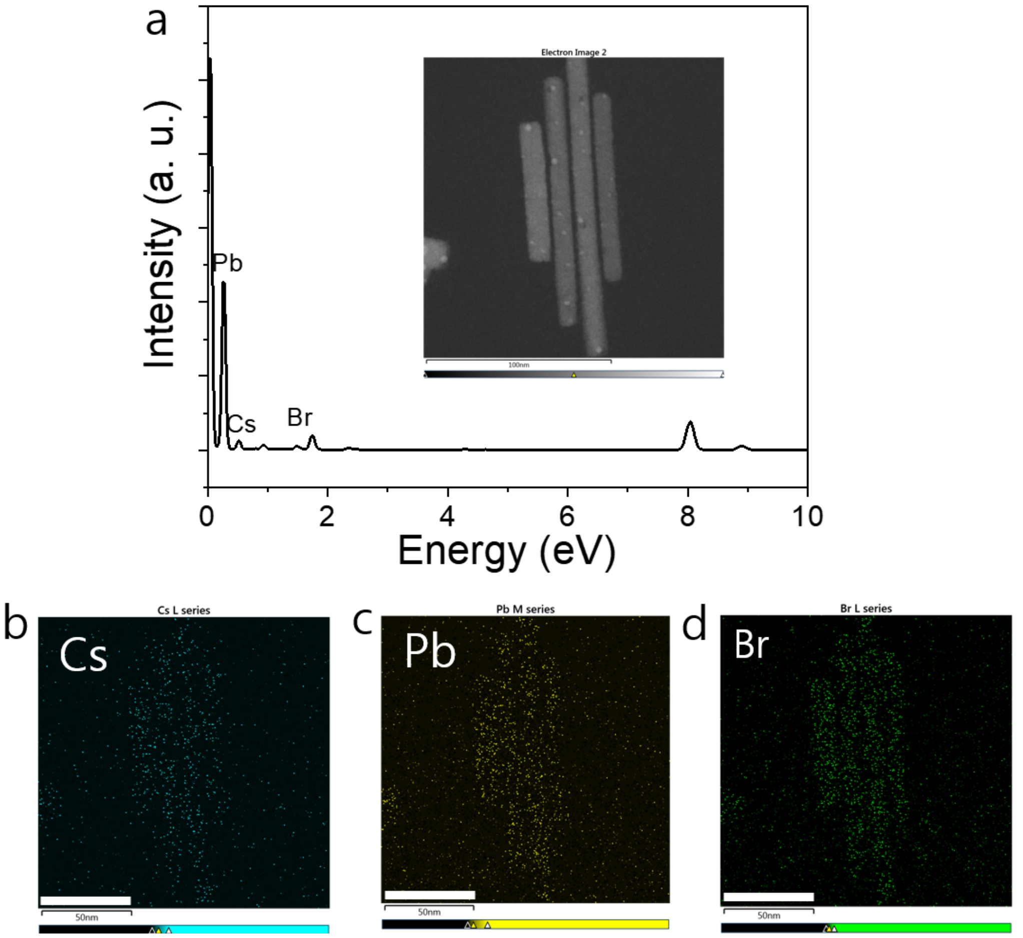

Figure S2. EDX and its images. (a) EDX spectrum of the aligned $CsPbBr_3$ NWs. The inset shows a scanning electron microscope (SEM) image of the nanowires, highlighting their aligned arrangement. Scale bar: 50 nm. b) to d) EDX elemental maps showing the distribution of (b) Cs (cyan), (c) Pb (yellow), and (d) Br (green) within the nanowires, indicating a uniform distribution of these elements along the nanowires. Scale bar: 50 nm. Quantitative EDS analysis shows atomic concentrations of Cs: 19.07%, Pb: 17.44%, and Br: 63.49%, confirming the $CsPbBr_3$ stoichiometry within experimental uncertainty (theoretical: Cs: 20%, Pb: 20%, Br: 60%). The structural uniformity ensures that the electronic and optical characteristics of each nanowire segment are similar, facilitating coherent dipole interactions necessary for SF.

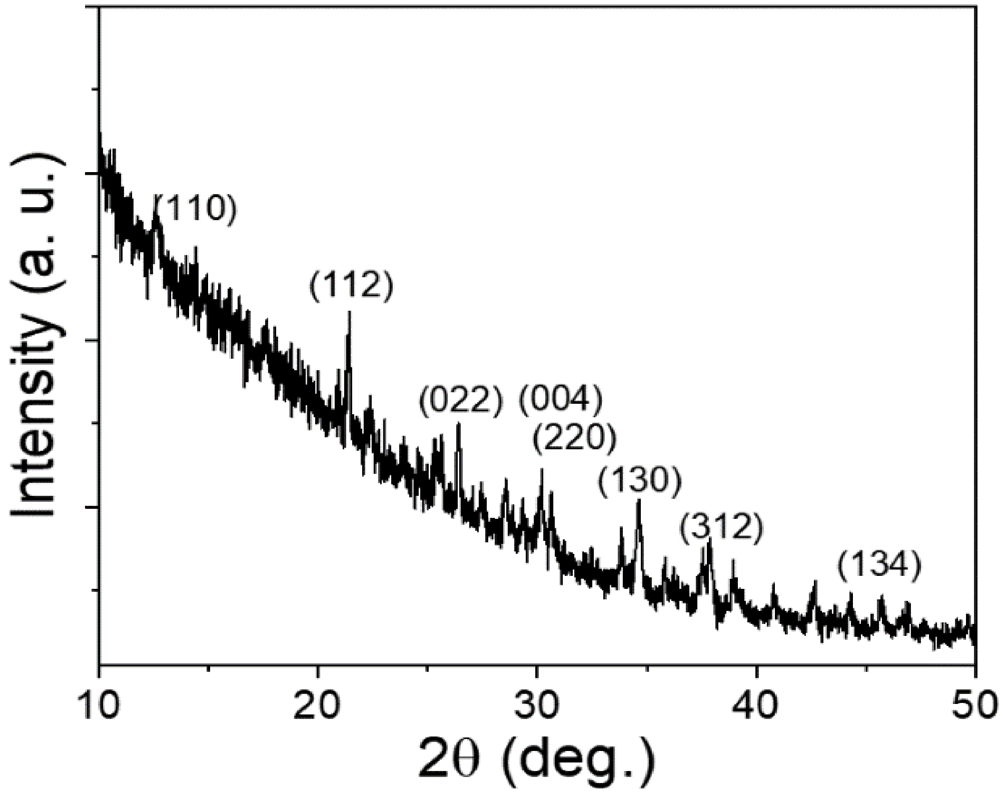

Figure S3. Powder XRD pattern showing characteristic diffraction peaks corresponding to the cubic perovskite phase of $CsPbBr_3$. Despite the limited signal intensity due to small sample quantities, the observed peak positions at $2\theta \approx 15.2°$ (110), 21.5° (112), 25.4° (022), 26.0° (004), 30.6° (220), 34.1° (130), 37.8° (312), and 43.2° (134) are consistent with the cubic $CsPbBr_3$ structure. The powder XRD data complement the high-resolution TEM lattice imaging (Figure 1a) and EDX compositional analysis (Figure S2) to confirm the crystalline phase essential for collective emission behavior. The relatively broad peaks and elevated background reflect the nanoscale dimensions and limited sample quantity, which are typical characteristics of solution-synthesized perovskite nanowires prepared as powder samples.

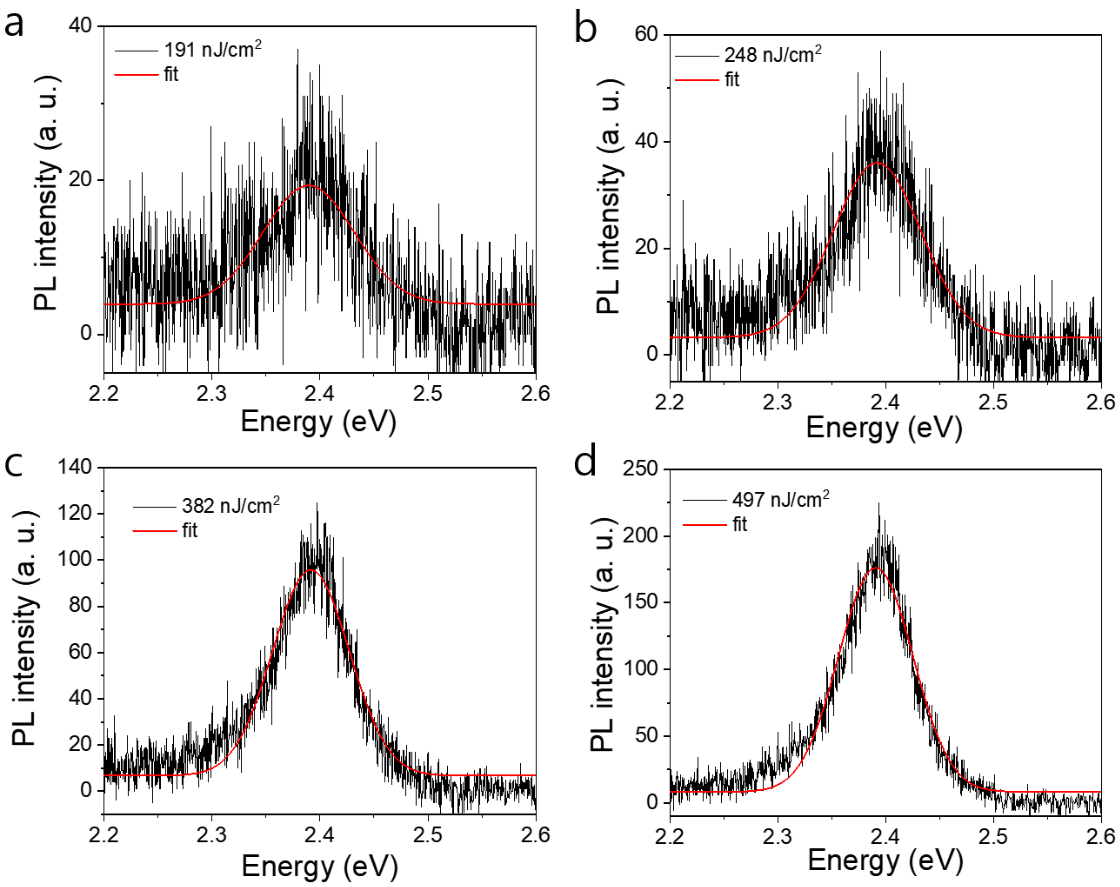

Figure S4. The black lines represent the experimental PL intensity at different excitation fluences. (a) 191 nJ/cm^2, (b) 248 nJ/cm^2, (c) 382 nJ/cm^2, and (d) 497 nJ/cm^2. The red lines are the Gaussian fits to the data. The Gaussian fitting helps in analyzing the integrated PL intensity, demonstrating the effect of varying excitation fluence on the PL properties of the nanowires.

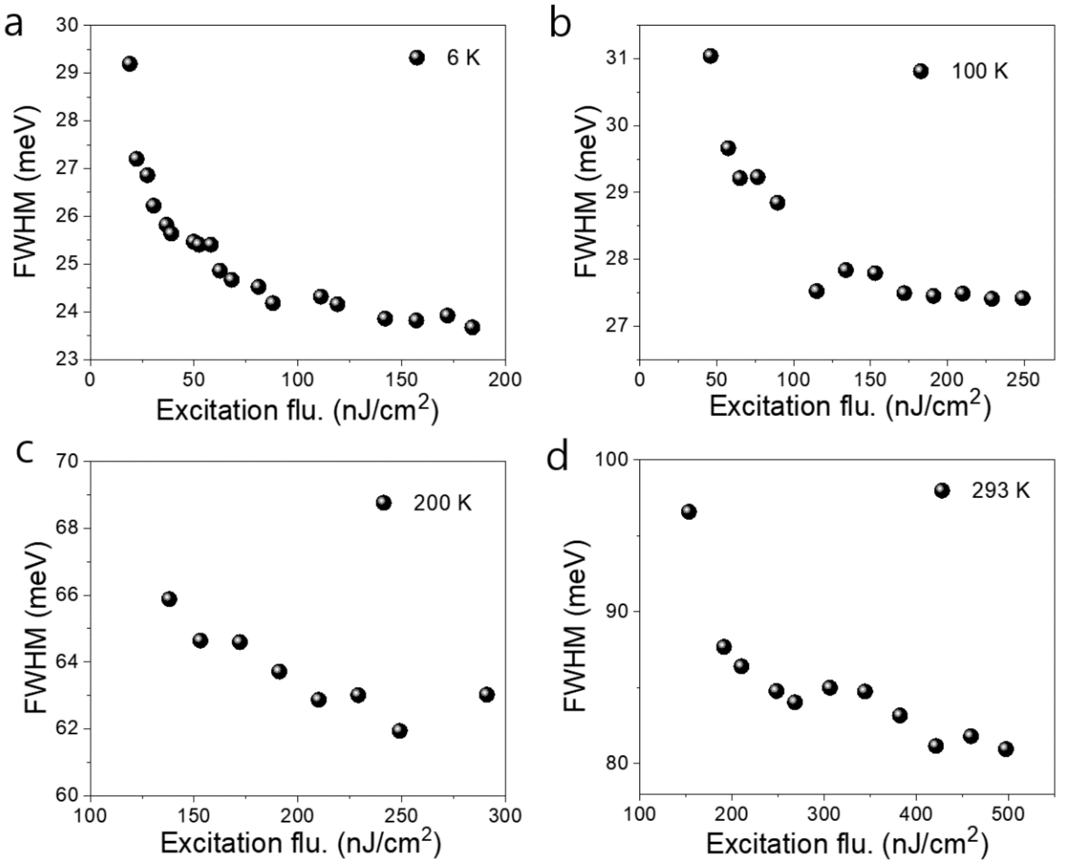

Figsure S5. Excitation fluence dependence of PL spectral linewidth (FWHM) at different temperatures. FWHM values of $CsPbBr_3$ NWs as a function of excitation fluence at (a) 6 K, (b) 100 K, (c) 200 K, and (d) 293 K. The systematic narrowing of the spectral linewidth with increasing excitation fluence indicates a transition from inhomogeneous broadening to a phase-coherent emission state. At each temperature, the linewidth decreases significantly as the excitation fluence approaches and exceeds the threshold value (P_{th}), consistent with the onset of CQE. The absolute FWHM values increase with temperature due to enhanced phonon-induced dephasing, but the narrowing phenomenon persists even at room temperature, demonstrating robust coherent interactions among Wannier-Mott excitons under ambient conditions.

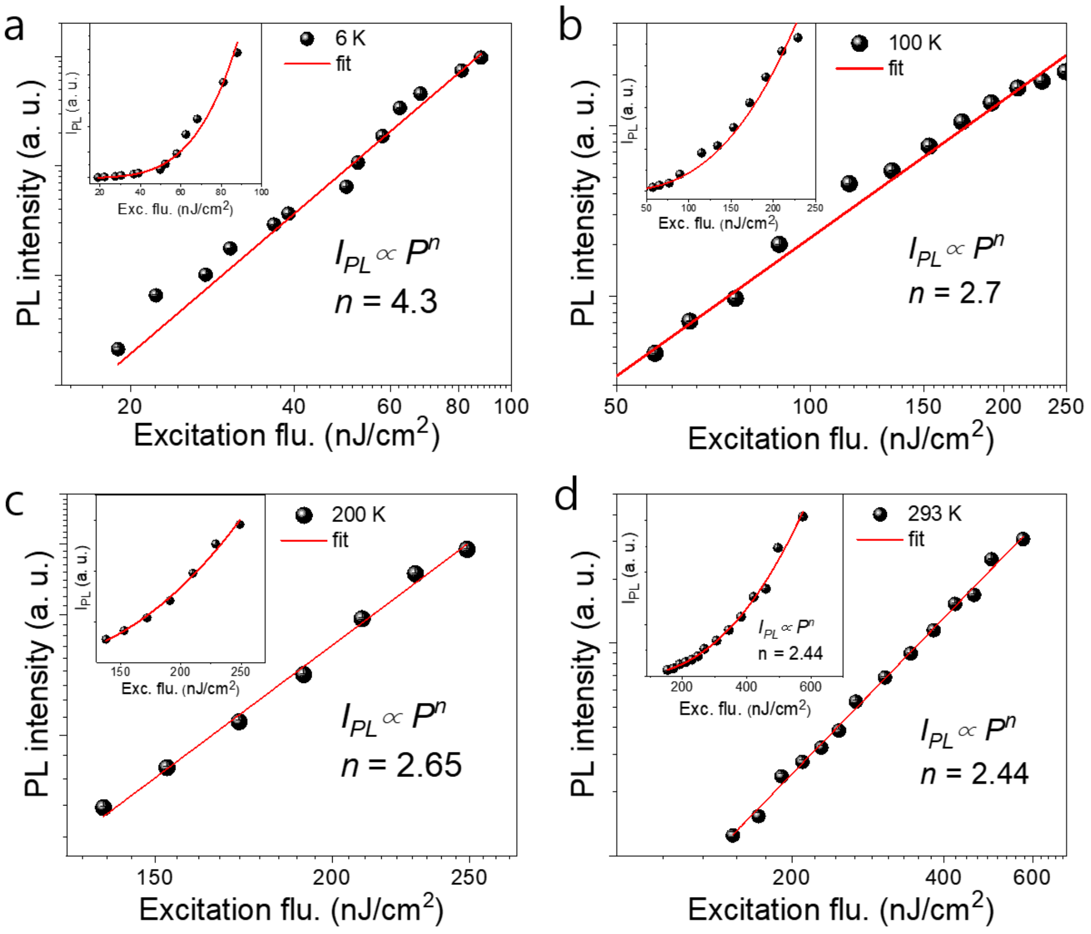

Figure S6. Temperature-dependent power-law scaling of PL intensity in $CsPbBr_3$ NWs. Log–log plots of integrated PL intensity versus excitation fluence at (a) 6 K, (b) 100 K, (c) 200 K, and (d) 293 K reveal superlinear behavior following a power-law dependence (I_{PL} α P^n), with exponents decreasing from $n = 4.3$ at 6 K to $n = 2.44$ at RT. Insets show linear-scale plots emphasizing the nonlinear emission threshold. The high power-law exponents, especially at low temperatures, are consistent with CQE phenomena such as SF, where multiple dipoles interact coherently to produce enhanced emission. Even at RT, the persistence of $n > 2$ suggests robust quantum coherence within the nanowire ensemble. Note: Although the extracted n values imply an increasing number of coupled emitters, they do not directly correspond to spectral linewidth narrowing (e.g., FWHM α $1/\sqrt{\boldsymbol{N}}$ from exchange narrowing theory). In our quasi-1D system, phonon-induced dephasing, static disorder (e.g., bundling), and ensemble inhomogeneity obscure such narrowing. Moreover, n reflects time-integrated PL scaling, whereas FWHM characterizes frequency-domain coherence. These quantities probe different aspects of coherence and are not expected to exhibit a direct one-to-one relationship.

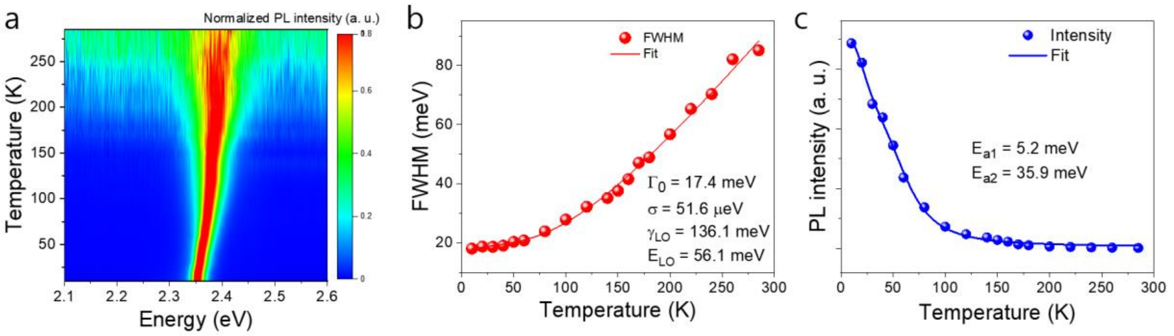

Figure S7. Temperature-dependent PL characteristics of $CsPbBr_3$ nanowire ensembles measured under a fixed excitation fluence of 382 nJ/cm^2. (a) False-color map of normalized PL spectra from 6 K to 300 K, showing gradual redshift and thermal quenching with increasing temperature. (b) Temperature dependence of the full width at half maximum (FWHM) of the PL peak, fitted using a phonon-assisted broadening model: $\Gamma(T) = \Gamma_0 + \sigma T + \gamma_{LO}/[\exp(E_{LO}/kT) - 1]$, yielding Γ_0 = 17.4 meV (inhomogeneous broadening) and E_{LO} = 56.1 meV (LO phonon energy). (c) Integrated PL intensity as a function of temperature, fitted with a biexponential Arrhenius model: $I(T) \propto 1 / [1 + A_1\exp(-E_{a1}/kT) + A_2\exp(-E_{a2}/kT)]$, revealing two thermally activated nonradiative pathways with activation energies of E_{a1} = 5.2 meV and E_{a2} = 35.9 meV.

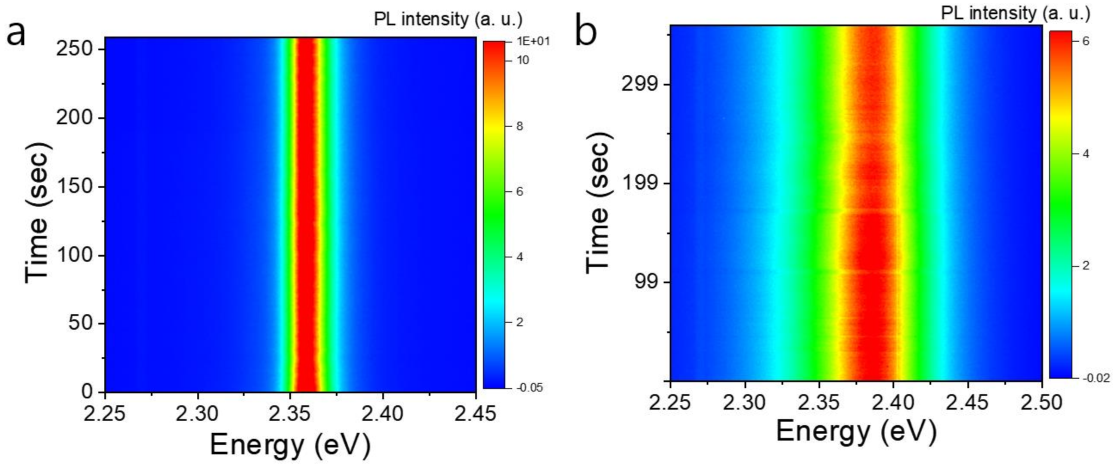

Figure S8. Photoluminescence time serious. (a) 6 K and (b) 293 K. The PL intensity is plotted as a function of energy and time, revealing stability and coherence at different temperatures. At 6 K, the PL spectrum shows a narrow, intense peak centered at ~2.35 eV, stable over time, indicating high coherence and minimal dephasing due to reduced phonon interactions. The high PL intensity suggests minimal defects and efficient, coherent light emission. In contrast, at 293 K, the PL spectrum broadens, with reduced intensity, reflecting increased thermal fluctuations and phonon interactions. This broadening points to enhanced dephasing and non-radiative recombination at room temperature. The stable, intense emission at 6 K emphasizes the nanowires potential for quantum light sources and low-threshold lasers, where high coherence and minimal dephasing are essential.

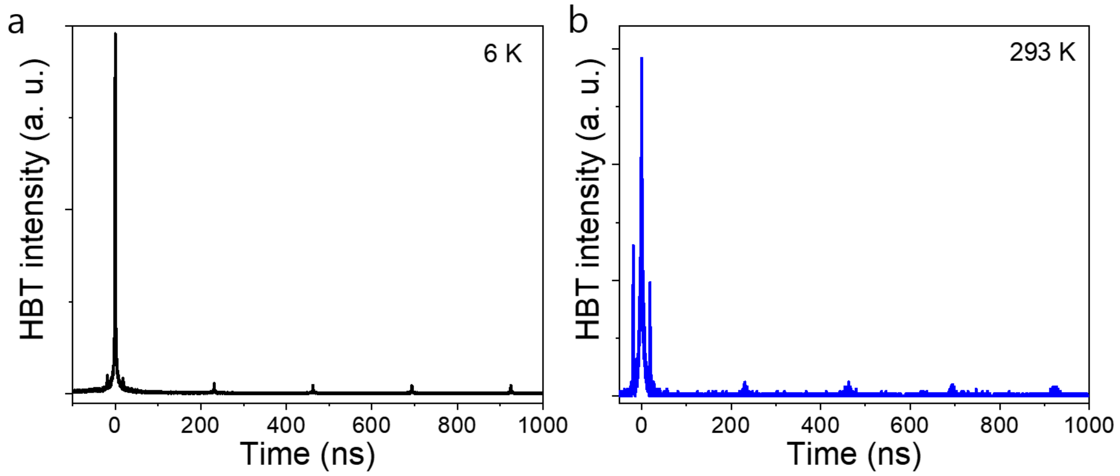

Figure S9. HBT measurements. Raw time-delay histogram acquired under pulsed excitation using the Hanbury Brown-Twiss setup. No normalization has been applied to the coincidence counts. The second-order correlation function shown in Fig. 5 of the main text was calculated from this dataset by normalizing to the average coincidence level at long time delays ($|\tau| >> 10$ ns), thereby yielding $g^2(\tau)$. (a) 6 K showing a sharp peak at zero delay. (b) at 293 K showing a broader and less intense peak at zero delay. The repetition rate is 4.3 MHz. The peaks on both sides of $t = 0$ are due to self-fluorescence from the APDs caused by the fiber coupled beam splitter. At 6 K, the intensity correlation function shows a sharp peak at zero delay, indicative of photon bunching. The high peak intensity at zero delay suggests a high degree of temporal coherence, which is typical for bunching or quantum emitters. This is consistent with the behavior of quantum-confined systems where thermal excitations are minimized, leading to enhanced coherence and stability of the emitted photons. In contrast, at 293 K, the intensity correlation function also shows a peak at zero delay, but it is significantly broader in time and reflects weaker temporal coherence compared to the low-temperature measurement, even though the normalized peak height remains similar. The broader peak indicates a reduction in temporal coherence, which can be attributed to increased phonon interactions and other non-radiative processes that are more prevalent at higher temperatures. These interactions cause dephasing and faster decay of the emission, leading to a broader and less pronounced correlation peak.

References

[1] F. Haake, H. King, G. Schröder, J. Haus, R. Glauber, Fluctuations in superfluorescence. *Phys. Rev. A* **1979**, 20, 2047.

[2] D. Ferreira, R. Bachelard, W. Guerin, R. Kaiser, M. Fouché, Connecting field and intensity correlations: The Siegert relation and how to test it. *American J. Phys*. **2020**, 88, 831.

[3] A. Ahlrichs, B. Sprenger, O. Benson, Photon counting and timing in quantum optics experiments. **2015**, Springer Series on Fluorescence: Adv. Photon Count.: Appl., Meth., Instrumen., 15, 319.

[4] R. H. Brown, R. Q. Twiss, Correlation between photons in two coherent beams of light, *Nature* **1956**, 177, 27.

[5] R. H. Brown, R. Q.T wiss, A test of a new type of stellar interferometer on Sirius, *Nature* **1956**, 178, 1046.

[6] M. R. Kar, U. Patel, S. Bhaumik, Highly stable and water dispersible polymer-coated $CsPbBr_3$ nanocrystals for Cu-ion detection in water, *Mater. Adv.* **2022**, 3, 8629.
